# Plant Alkaloids as Antiplatelet Agent: Drugs of the Future in the Light of Recent Developments

**DOI:** 10.3389/fphar.2016.00292

**Published:** 2016-09-22

**Authors:** Qurrat-Ul- Ain, Haroon Khan, Mohammad S. Mubarak, Aini Pervaiz

**Affiliations:** ^1^Department of Pharmacy, Abdul Wali Khan University MardanMardan, Pakistan; ^2^Department of Chemistry, The University of JordanAmman, Jordan

**Keywords:** alkaloids, plants, platelet aggregation, homeostasis, future drugs

## Abstract

An alkaloid is a class of naturally occurring organic nitrogen-containing compounds that are frequently found in the plant kingdom. Many alkaloids are valuable medicinal agents that can be utilized to treat various diseases including malaria, diabetics, cancer, cardiac dysfunction etc. Similarly, platelet aggregation beyond the purpose of homeostasis is the underlying cause of blood clotting related diseases. This review presents a thorough understanding of alkaloids as antiplatelet agents with a possible mechanism of action based on the literature of the last decade. In addition, this review will address the antiplatelet activity of alkaloids and their medicinal usage as potent antiplatelet agents with a description of structural relationship activity and possible lead compounds for future drug discovery.

## Introduction

Platelets (thrombocytes) are small irregularly shaped anuclear cells. The resting platelets circulate in the blood in discoid shape which is changed upon activation. When blood vessels are damaged or injured, platelets rush and form aggregates at the site of injury to stop the bleeding and this is facilitated by their binding to the exposed activated thrombin receptor (Warfarin Antiplatelet Vascular Evaluation Trial Investigators et al., 2007; Holmes Jr et al., 2009). Blood platelets are involved in normal homeostasis. The normal homeostatic system limits blood loss by regulated interaction between components of the vessel wall, circulating blood platelets and plasma proteins. Platelets play an important role in the development of cardiovascular diseases. Arterial thrombosis is an acute complication that develops on chronic lesions of atherosclerosis that results in a heart attack and stroke. These chronic inflammatory processes are the central pathophysiological mechanism by which lipid accumulation provides substrates for occlusive thrombus formation (Jackson et al., 2003; Aradi et al., 2013). Some drugs used to treat inflammation may cause the undesired side effect of suppressing normal platelet function, therefore, there is always a need for alternative medicines to deal with such cases.

It is estimated that about 80% of the population of developing countries meet their primary health care needs mainly through plant-based traditional healing (Amin and Khan, 2016). Different parts of medicinal plants, rarely the whole plant, are mostly used in the preparation of traditional medicines (Khan, 2014a,b). For so many years, despite criticisms, traditional medicine has gained tremendous revival. Not only traditional healers provide immediate health care to the rural population, but also play an important role in providing leads to the discovery of pharmacologically active plant-derived compounds (Butler, 2008; Khan and Rauf, 2014).

Alkaloids are small organic molecules, secondary metabolites of plants, containing nitrogen usually in a ring; about 20% of plant species consist of alkaloids (Amirkia and Heinrich, 2014; Khan, 2016a). Alkaloids are mainly involved in the plant defense against herbivores and pathogens. They are pharmaceutically significant, traditional and modern uses of alkaloids are 25 to 75% in drugs, indicating their great therapeutic potential (Khan, 2016b; Pervez et al., 2016). The basic character of alkaloids is no longer a pre-requisite for an alkaloid and the chemistry of nitrogen atoms allows at least four groups of nitrogenous compounds. Some synthetic compounds of similar structures are also termed as alkaloids (Khan et al., 2016). Some alkaloids are free bases while others form salts with organic acids such as oxalic and acetic acids. Some plant alkaloids are present in a glycosidic form such as solanine in solanum. The Alkaloid biosynthesis pathway is specifically involved in the decarboxylation of compounds (Grynkiewicz and Gadzikowska, 2008). Medicinal plants are a rich source of alkaloids having antiplatelet and anticoagulant activities. The commonly used antiplatelet, aspirin, originated from salicin obtained from willow plant commonly used in pain medication. This review describes various alkaloids isolated from plant sources having antiplatelet activity with possible mechanisms and candidates for further detailed studies in the drug discovery as antiplatelet agents.

## Alkaloids as antiplatelet agents

In addition to synthetic drugs, several alkaloids have been utilized as antiplatelet agents (Table 1). Rutaecarpine, an alkaloid isolated from *Evodia rutaecarpa*, exhibited significant antiplatelet activity which was further augmented by its derivatives, 2,3-methylenedioxyrutaecarpine, 3-chlororutaecarpine and 3-hydroxyrutaecarpine by interfering with different mediators of clot formation (Sheu et al., 1996; Son et al., 2015). This change in activity was attributed to hydroxyl and methoxy groups substitution. Park along with his Korean research fellows isolated four acid amides (piperine, pipernonaline, piperoctadecalidine, and piperlongumine) from *Piper longum* L. (Park et al., 2007). The isolated compounds evoked marked antiplatelet effect in a concentration-dependent manner. Of the isolated lot, piperlongumine was most potent by acting through multiple ways. Later on, piperlongumine was used as a lead compound and its various derivatives were synthesized and exhibited a potential inhibitory effect on washed rabbit platelet aggregation induced by collagen, arachidonic acid (AA), and platelet activating factor (PAF). On the other hand, 1-(3,5-dimethylpiperidin-1-yl)-3-(3,4,5-trimethoxyphenyl)prop-2-en-1-one was reported to have the most inhibitory effect on platelet aggregation induced by collagen. This compound showed 100% inhibition at a concentration of 150 and 300 μM (Park et al., 2008). In addition, a group of researchers in 2010 extracted several alkaloids including veratroylgermine from *Veratrum dahuricum*. This compound was found to produce the strongest inhibition of the platelet aggregation induced by arachidonic acid with an inhibition rate of 92.0% at 100 μM (Tang et al., 2010). Similarly, Li et al. (2002) obtained six alkaloids including spiramine C1 from *Spiraea japonica*. This diterpene alkaloid was reported to inhibit PAF-induced platelet aggregation (Li et al., 2002). Moreover, spiramine C1 inhibited platelet aggregation induced by PAF, ADP, and arachidonic acid with IC_50_ values of 30, 56, and 29.9 μM, respectively, in a concentration-dependent manner; suggesting a non-selective antiplatelet aggregation action. In addition, the inhibitory effect of spiramine C1 on arachidonic acid was comparable to that of aspirin suggesting a strong potential of this compound as a lead in the drug discovery against platelet aggression.

**Table 1:** **The alkaloids isolated from plant with antiplatelet activities**.

**S. No**	**Plant name**	**Chemical structure**	**References**
**1**	*Evodia rutaecarpa*	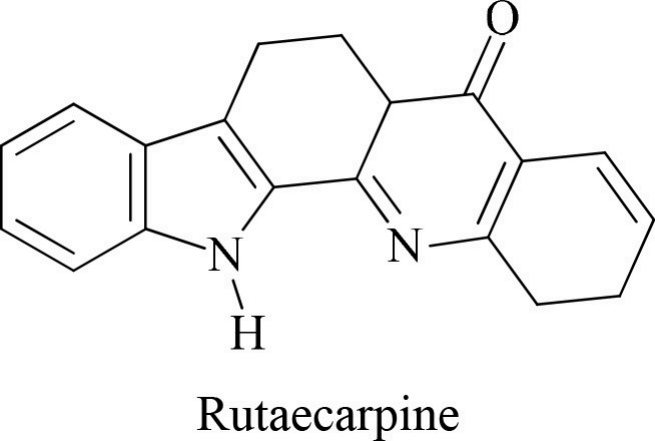	Asahina and Mayeda, 1916; Son et al., 2015
**2**	*Piper longumine*	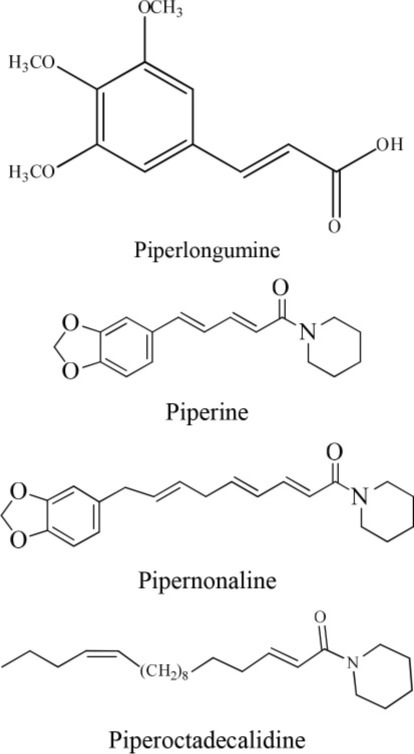	Park et al., 2007
3	*Veratrum dahuricum*	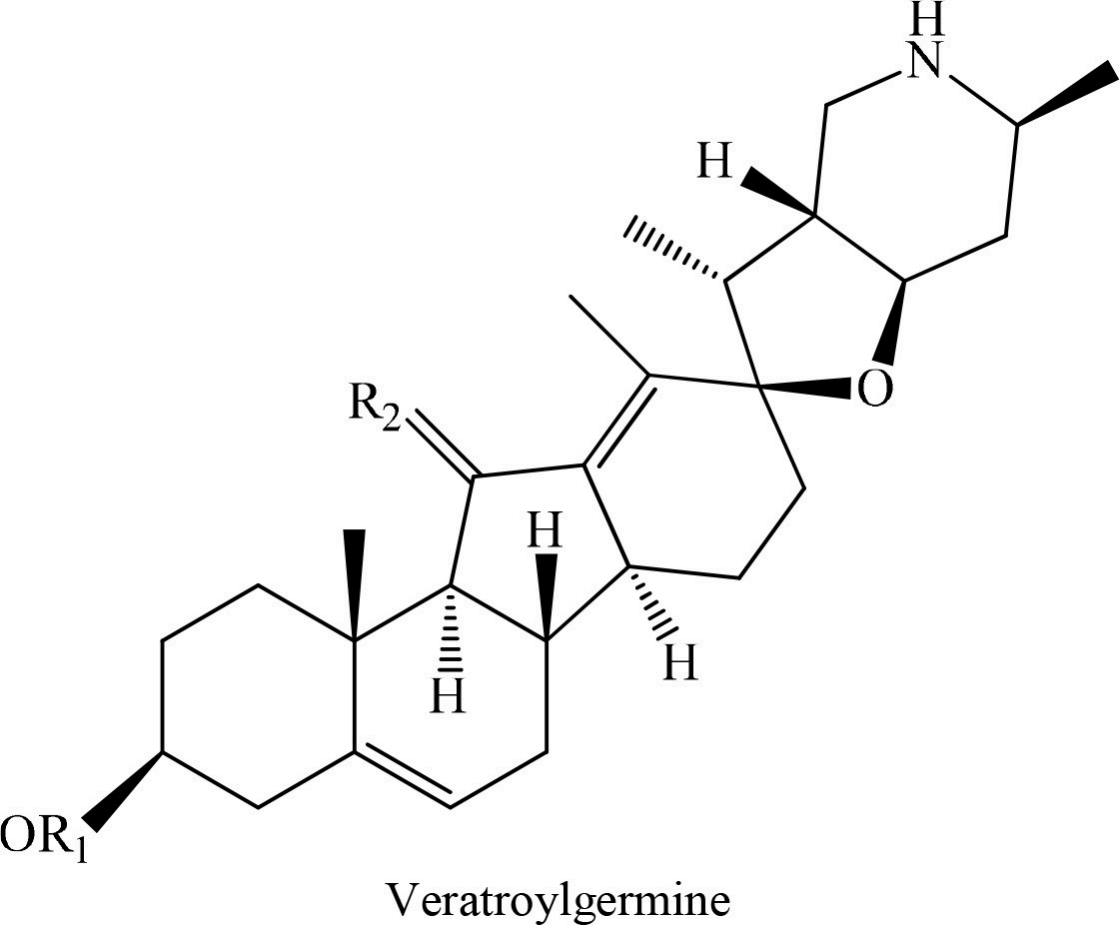	Tang et al., 2010
4	*Spiraea japonica*	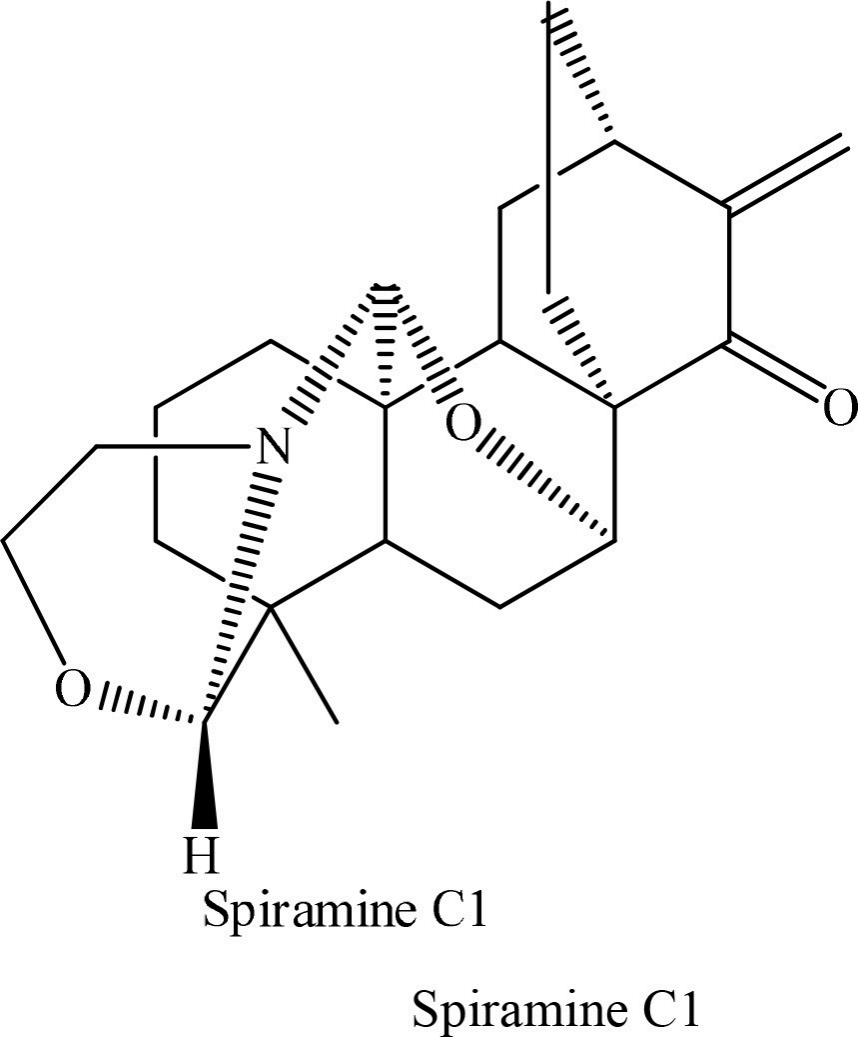	Li et al., 2002
5	*Perganum harmala*	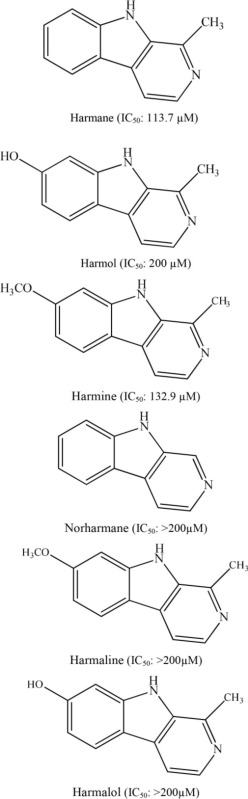	Im et al., 2009
6	*Rollinia mucosa*	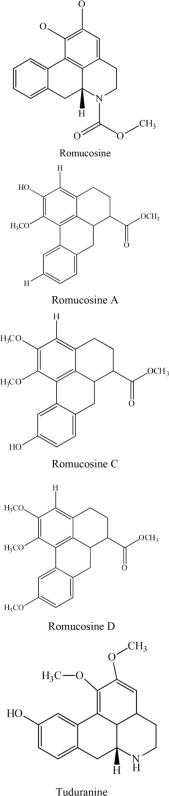	Kuo et al., 2001, 2004
**7**	*Leonurus sibiricus*	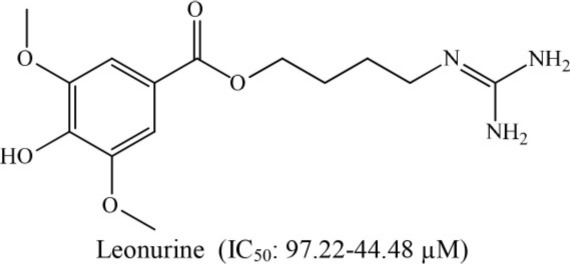	Lin et al., 2007
8	*Cassytha filiformis* L	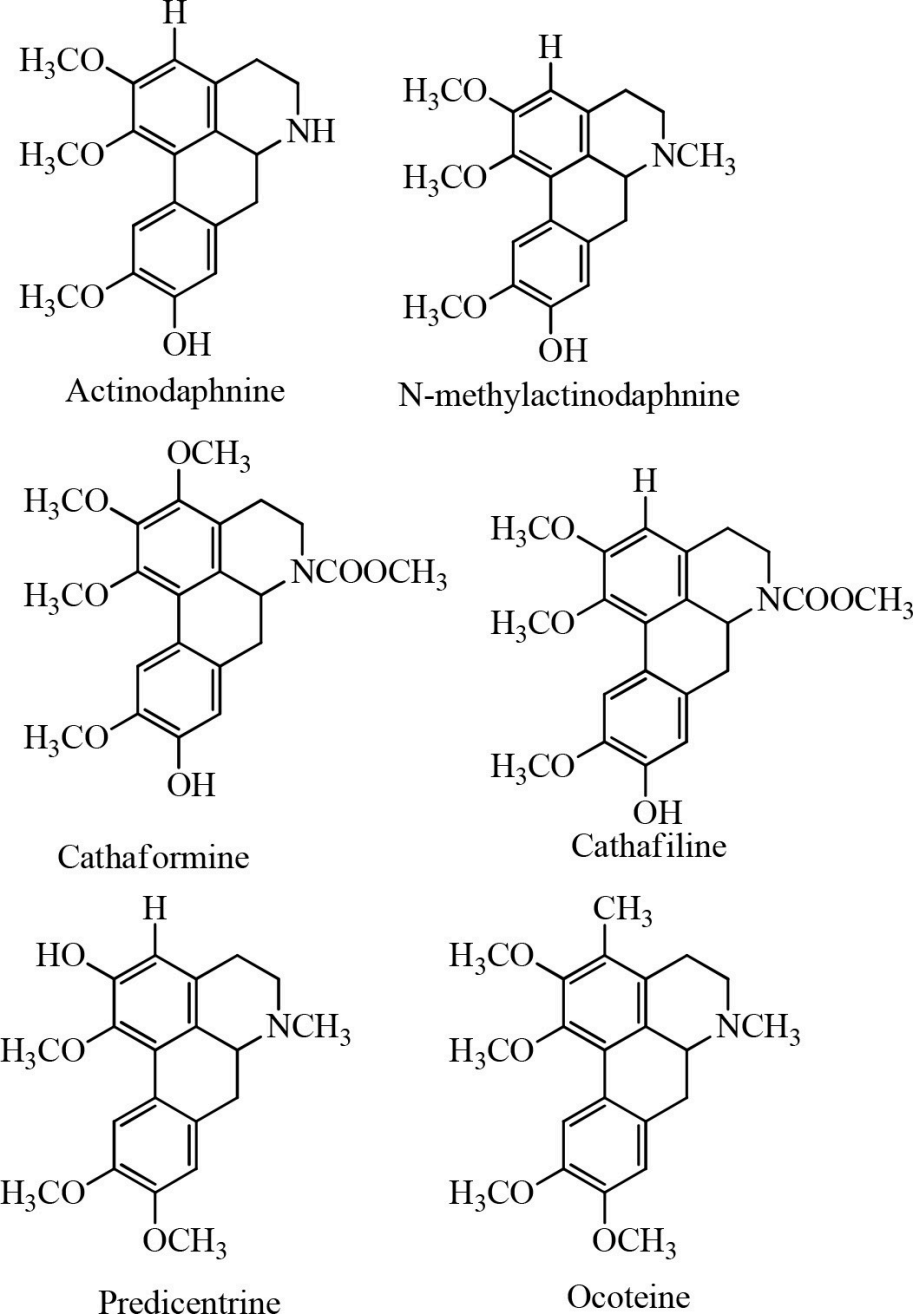	Wu et al., 1998
9	*Hernandia nymphaefolia*	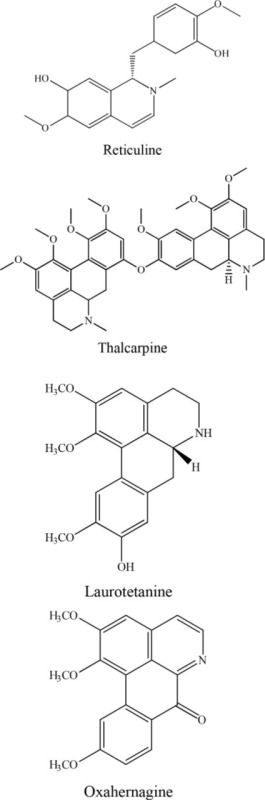	Chen et al., 2000
10	*Rauwolfia serpentine*	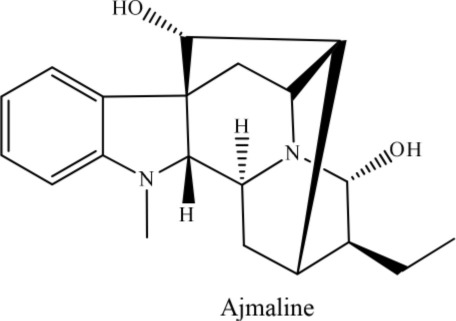	Rahman et al., 1991
11	*Carcuma aromatic*	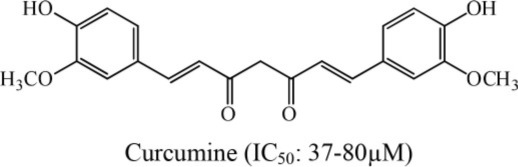	Jantan et al., 2008
12	*Illigera luzonensis* Merr	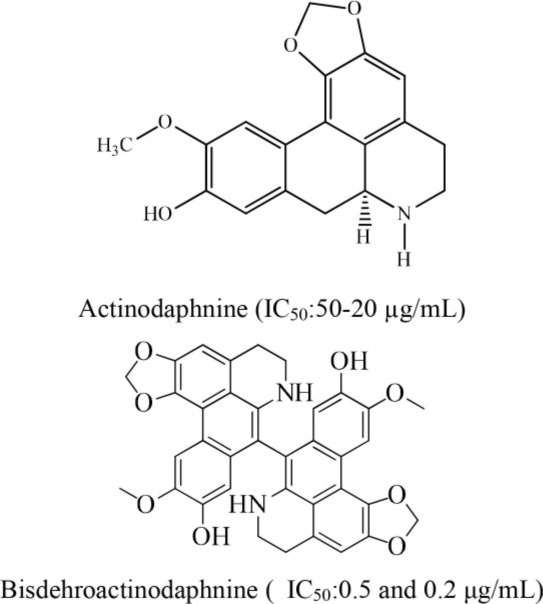	Huang et al., 2014
13	*Melicope semecarpifolia*	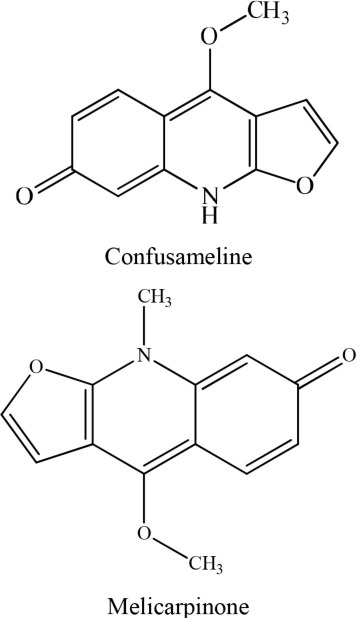	Chen et al., 2001
14	Berberis	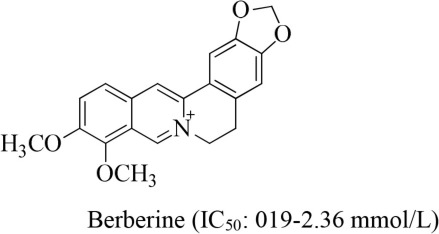	Chu et al., 1996
15	*Ruta graveolens*	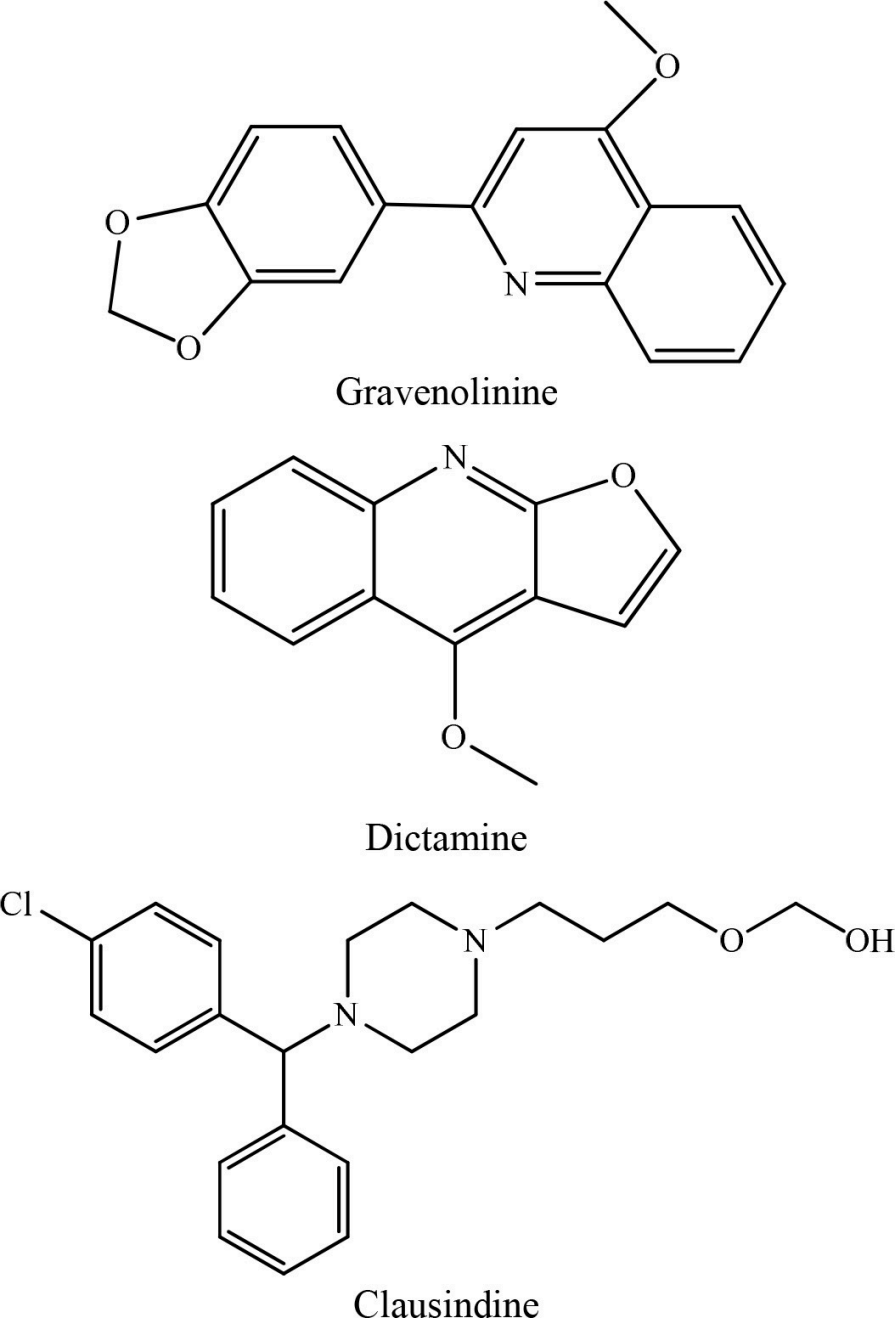	Wu et al., 2003

In 2009, a group of researchers discovered that β-carboline alkaloids obtained from *Preganum harmala* possess good antiplatelet activity. Among the isolated β-carboline alkaloids, harmane and harmine were the most potent with IC_50_ values of 113.7 and 132.9 μM, respectively, whereas harmol possessed medium potency with an IC_50_ value of 200 μM (Im et al., 2009). In terms of structure-activity relationship on these compounds' inhibition of platelet aggregation, it is assumed that the double bond in the C4–C9 position of the tricyclic aromatic structure confers a basal inhibition on collagen-induced platelet aggregation, as harmaline and harmalol showed the weakest antiplatelet activity, both of which lack such a double-bond. Introducing a methyl group at the C13 position could strengthen such an inhibition as norharmane was much weaker than harman. In addition, the antiplatelet potency varied with the nature of groups at C1 position.

Similarly, Kuo and colleagues found that romucosine, romucosine A, romucosine C, romucosine D and tuduranine, phenanthrene type of alkaloids, extracted from *Rollinia mucosa* Baill possessed a good antiplatelet activity (Kuo et al., 2001, 2004). It has been observed that methoxyl substitution of C-10 enhanced the activity more than hydroxyl substitution.

On the other hand, leonurine, isolated from the aerial part of *Leonurus sibircus* var. albiflora by Hang and coworkers, significantly inhibited rabbit platelet aggregation induced by thrombin, arachidonic acid, and collagen *in vitro* with IC_50_ values of 97.22, 31.03, and 44.48 μM, respectively (Lin et al., 2007). Furthermore, the methanol extract of *Cassytha filiformis* was found to contain cathafiline, cathaformine, actinodaphnine, predicentrine, and ocoteine. These alkaloids exhibited remarkable vasorelaxant and inhibitory effects on the platelet aggregation in washed rabbit platelets induced by ADP (20 μm), arachidonic acid (100 μM), collagen (10 μM), and PAF (3.6 nM), respectively. All six alkaloids showed antiplatelet effects to varying extent (Wu et al., 1998). In a paper published by Chen et al. (2000), it was reported that the trunk bark of *Hernandia nymphaefolia* possessed strong antiplatelet aggregation *in vitro* due to the presence of six alkaloids: laurotetanine, oxohernagine, thallicarpine, reticulline, vateamine, and hernandaline. The most potent alkaloid was oxohernagine with 90% inhibition whereas laurotetanine exhibited 87% inhibition at a concentration of 100 μM. On the other hand, thalicarpine caused cell lysis at a concentration of 100 μM and showed 65% inhibition of platelet aggregation (Chen et al., 2000). Rahman et al. (1991) showed that ajmaline and acetyl ajmaline present as minor alkaloids in the *Rauvolfia serpentina* selectively inhibited PAF-induced aggregation in a concentration-related manner. Similarly, ajmaline or acetyl ajmaline also inhibited the lethal effects of PAF in rabbits; PAF (8–11 μg/kg i.v.) caused sudden death in rabbits due to platelet aggregation and cardiac failure (Rahman et al., 1991; Unnikrishnan and Nishteswar, 2015).

In an investigation conducted by Jantan et al. (2008), curcumin was isolated from *Curcuma Aromatica*. This alkaloid showed strong inhibition of platelet aggregation induced by arachidonic acid with an IC_50_ value of < 84 μM. Moreover, curcumin was the most effective antiplatelet compound as it inhibited arachidonic acid, collagen, and ADP-induced platelet aggregation with IC_50_ values of 37.5, 60.9, and 45.7 μM, respectively (Jantan et al., 2008). Similarly, Huang et al. (2014) reported that aporphine alkaloids are the primary components of *Illigeralu zonensis*, exhibited varying degrees of antiplatelet activity effect. However, bisdehydroaporphine and actinodaphnine were most potent alkaloids (Huang et al., 2014). Furthermore, a study carried out by Wu et al. (2003) on *Rutagra graveolens*, a plant belonging to family Rutaceae revealed the presence of dictamine, chalepensin, clausindin, and graveolinine; these compounds displayed significant inhibition of platelet aggregation (Wu et al., 2003). On the other hand, Huang et al. (2014) discovered that the plant *Illigera luzonensis* Merr contains aporphine alkaloids. These researchers also found that actinodaphnine, which belongs to aporphine alkaloids, exhibited significant inhibition of the aggregation of washed rabbit platelets with an IC_50_ value in the range of 50–20 μg/mL (Huang et al., 2014). Furthermore, Chen et al. (2001) isolated three new quinoline alkaloids from the root bark of Melicope semecarpifolia along with 26 known compounds. Several of these isolated compounds exhibited significant antiplatelet inhibition against arachidonic acid-induced aggregation, collagen-induced, and PAF-induced aggregation (Chen et al., 2001).

Altogether, these findings showed the potential of plant alkaloids as antiplatelet agents or lead compounds. Most recently, Lee et al. (2016) isolated and characterized alkaloids from *Scolopendra subspinipes mutilans*, a product registered in various Pharmacopeia (Lee et al., 2016). The isolated alkaloids showed antithrombotic and antiplatelet activities *in vitro* and *in vivo*, so that they appeared as strong candidates or lead compounds. However, detailed studies are suggested for the reported plant-alkaloids discovery of molecules of clinical utility. A Taipei research group in 2003 isolated various components from *Ruta graveolens* including clausindin, dictamine, and graveoline. they showed strong antiplatelet activity against various mediators of clot formation (Wu et al., 2003). Different structural parameters of these alkaloids were unable to describe structural relationship activity.

## Antiplatelet mechanism of plant alkaloids

**Figure 1:**
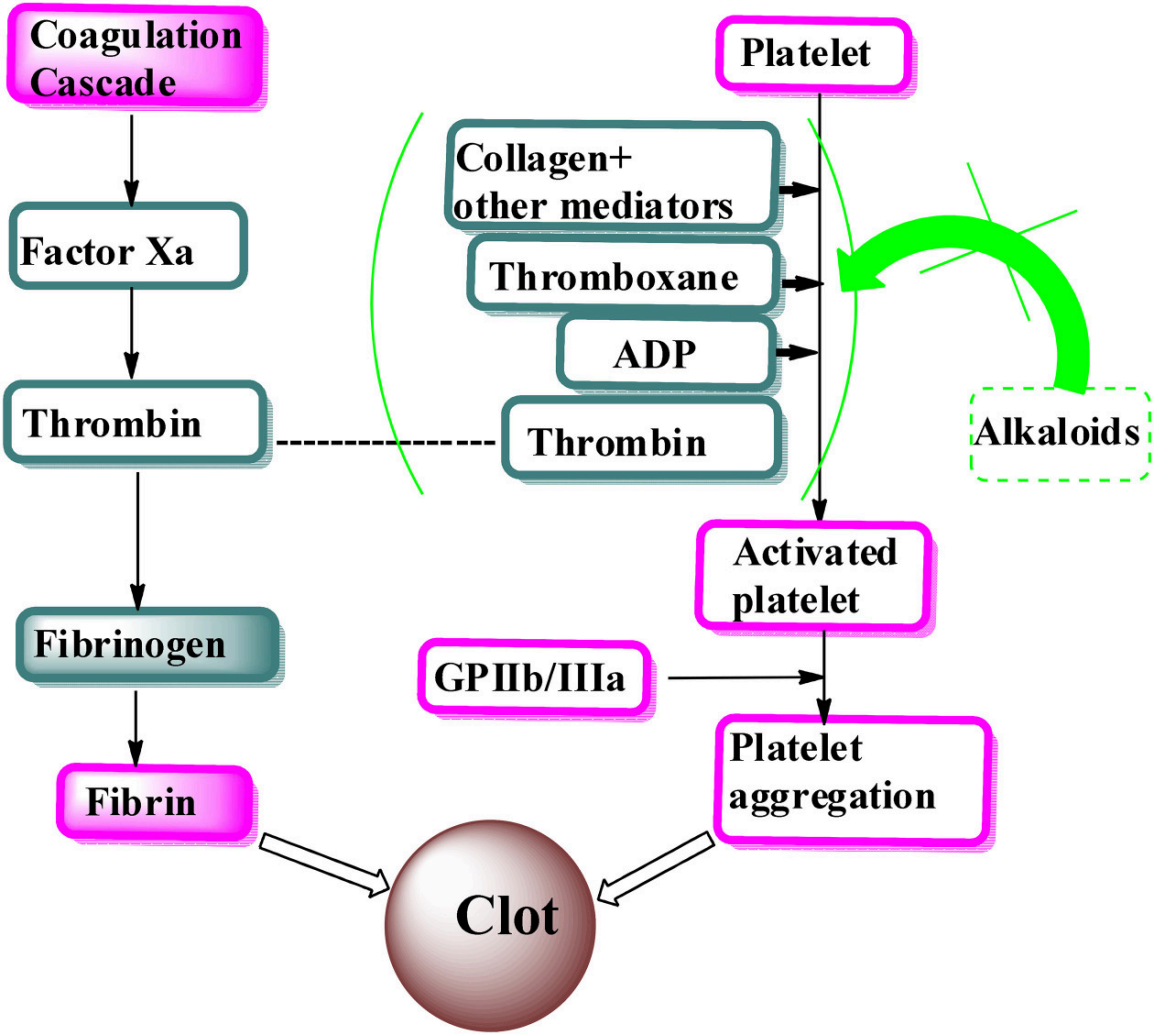
**Proposed mechanism of alkaloids as antiplatelet**.

Various mechanisms have been proposed for the antiplatelet activity of different isolated plant-alkaloids (Figure 1). Researchers have proposed that berberine significantly inhibited platelet aggregation by inhibiting synthesis of thromboxane A2 induced by adenine diphosphate, arachidonic acid, collagen (Chu et al., 1996). In addition, clausine-D, isolated from *Clausena excavate*, displayed antiplatelet effect by inhibition of thromboxane A2 formation. Higher concentration of clausine-D (150 μM) produced almost complete inhibition of collagen-induced platelet aggregation (Wu et al., 1994). Similarly, hernandaline and apomorphine alkaloids exhibited antiplatelet activity by complete inhibition of platelet aggregation induced by PAF at 50 μg/mL, whereas reticuline completely inhibited arachidonic acid and collagen-induced platelet aggregation (Chen et al., 2000). On the other hand, phylligenin alkaloid showed strong dose-dependent inhibitory activity. The β-carboline alkaloids selectively inhibited PLCgamma2 and protein tyrosine phosphorylation with sequential suppression of cytosolic calcium mobilization and arachidonic acid liberation (Im et al., 2009), indicating that harmane and harmine have a potential to be developed as novel agents for atherothrombotic diseases.

Based on the literature, the alkaloids isolated from various sources provoked antiplatelet activity generally mediated through multiple mechanisms, different from aspirin which is a cyclooxygenase inhibitor (Chen et al., 2000) and thus expressed their widespread potential as new effective agents of the class or as lead compounds.

## Conclusion

From the above literature review, it is concluded that alkaloids are present abundantly and in high concentrations in natural medicinal plants and several of them possess antiplatelet activity. Moreover, the most important and effective alkaloids found that can be used as antiplatelet agents are curcumin, reticulin, piperlongumine, and melicarpinone that can be used as antiplatelet agents. From a mechanistic point of view, they are very versatile and interfere with various mediators of clot formation, unlike aspirin which is a cyclooxygenase inhibitor. In this regard, these agents are special candidates for further detailed studies to ascertain their clinical utility and could be lead compounds for better antiplatelet activity.

## Review criteria

The original research articles were searched in Google scholar for keywords having the terms alkaloid and antiplatelet activity. Articles from the last decade were selected to prepare this review.

## Author contributions

Q-U-A, has written the initial draft; AP, drawn all the structures in MS; MM, contributed in the scientific writing of MS; HK, proposed the idea and finalized the MS.

### Conflict of interest statement

The authors declare that the research was conducted in the absence of any commercial or financial relationships that could be construed as a potential conflict of interest.
